# Potential common factors associated with predisposition to common cold in middle-aged and elderly Japanese: A community-based cross-sectional study

**DOI:** 10.1097/MD.0000000000010729

**Published:** 2018-05-18

**Authors:** Michi Shibata, Taizo Iwane, Ryoko Higuchi, Kaname Suwa, Kei Nakajima

**Affiliations:** a School of Nutrition and Dietetics, Faculty of Health and Social Services, Kanagawa University of Human Services; b Department of Nutrition, St. Marianna University School of Medicine Hospital, Kawasaki, Kanagawa; c Saitama Health Promotion Corporation, Yoshimimachi, Hikigun; d Department of Endocrinology and Diabetes, Saitama Medical Center, Saitama Medical University, Kawagoe, Saitama, Japan.

**Keywords:** body weight, common cold, constipation, diarrhea, lifestyles, sleep duration

## Abstract

People worldwide frequently catch a common cold, which occasionally develops into secondary severe conditions such as pneumonia. However, it is unclear whether predisposition to the common cold is associated with the individual's characteristics including age, body weight, lifestyles, diets, and intestinal functions, besides exposure to a responsible pathogen. We addressed this issue epidemiologically considering many relevant clinical factors.

We reviewed data from a cross-sectional study consisting of 39,524 apparently healthy Japanese aged 40 to 79 years (26,975 men and 12,549 women) who underwent a checkup in 2007. Self-reported predisposition to common cold (SPCC) and relevant clinical conditions and parameters were considered.

We observed no significant difference in most clinical parameters including age, body mass index (BMI), glycated hemoglobin (HbA1c), and prevalence of men and current smokers between subjects with and without SPCC. In univariate analysis, circulating white blood cell (WBC) count and serum alanine-aminotransferase (ALT) were significantly higher in subjects with SPCC than in those without, whereas serum high-density lipoprotein cholesterol (HDL-C) and duration of sleep were lower. In logistic regression analysis after full adjustment for relevant confounding factors, BMI categories except BMI of ≥27.0 kg/m^2^ were significantly associated with SPCC compared with BMI of 23.0 to 24.9 kg/m^2^. Short duration of sleep (≤5 hours), occasional alcohol drinking, and no-exercise were significantly associated with SPCC compared with 7 hours sleep duration, no-drinking alcohol, and low frequent exercise (twice per month), respectively. All gastrointestinal disorders (gastric complaints, constipation, and diarrhea) were independently associated with SPCC. Imbalanced diet and taking a snack were also associated with SPCC in a degree dependent manner. Furthermore, WBC count, serum ALT, and HDL-C (as continuous variables) were associated with SPCC (HDL-C was inversely), whereas no significant association was observed between SPCC and age, smoking, HbA1c, and pharmacotherapy for diabetes, hypertension, and dyslipidemia.

Our results demonstrated that multifactorial conditions and parameters might be simultaneously associated with the predisposition to common cold. Prospective studies including detailed common cold questionnaire and measurements are needed to confirm currently suspected causative and protective factors.

## 1 Introduction

Irrespective of remarkable progress in medicine, most people worldwide regardless of sex or age experience a common cold several times per year.^[1–4]^ It has been reported that about half of adult men in a northern district of Japan have experienced a common cold at least once per year.^[3]^ Although the common cold is mildly cured without severe complications and generally is not a life-threatening disease, it elicits systemic dysfunctions during the infection (e.g., fever, sore throat, headache, and cough) and occasionally leads to secondary severe conditions such as bacterial respiratory infection.^[1,4]^ A repeated common cold impairs quality of life and work productivity,^[1]^ with substantial economic loss for direct (treatments) and indirect (missed work) costs.^[4]^

In principal, avoiding exposure to the causative pathogen such as human rhinovirus is essential, which could be partly feasible by physical interventions such as handwashing, use of a face mask, and gargling.^[4]^ In addition, other protective or promotive factors also influence the onset and severity of the common cold because the susceptibility to the infection can vary inter- and intra-individually, owing to socioeconomic and emotional conditions.^[5–7]^ Presently, it has been reported that some factors may exert protective action including the intake of nutrients such as zinc and vitamin C and taking regular exercise,^[1,2,4]^ which both relate to immune function.^[8–10]^ Furthermore, diabetes and obesity, which are increasing worldwide,^[11–13]^ have been considered to increase the susceptibility to influenza, pulmonary infections, and severe episodes of pneumonia, which increase the risk for hospitalization.^[14–20]^

There is a lack of large epidemiological studies that have evaluated the associations between the predisposition of the common cold and the causative and protective factors. These include body weight as an overall reflector of the nutritional condition, the clinical/biochemical parameters that relate to cardiometabolic diseases, and the common gastrointestinal symptoms (gastric discomforts, diarrhea, constipation), which correlate with the microbiota and immune function.^[21–23]^ In this context, we have addressed these issues in a large epidemiological study of apparently healthy middle-aged and elderly people.

## 2 Methods

### 2.1 Study design and subjects

A cross-sectional investigation was undertaken using data recorded during medical checkups of people living or working in Saitama Prefecture, a suburb of Tokyo, Japan.^[24]^ We reviewed digitally recorded data for 116,817 subjects aged ≥18 years who underwent a medical health checkup in 2007 at the public interest corporation Saitama Health Promotion Corporation. After age restriction (40–79 years), 51,407 subjects remained for the study. Because the prevalence of lifestyle-related diseases such as type 2 diabetes and metabolic syndrome increases in Japanese people aged ≥40 years, who are nowadays supposed to undergo specific health checkup.^[25]^ Individuals who required immediate treatment for serious conditions, such as suspected cancer, heart failure, ischemic coronary disease, or infectious pneumonia, which were determined by physicians based on symptoms and laboratory data including chest x-rays, were excluded from the study. Additionally, individuals with a body mass index (BMI) of <15 kg/m^2^ or ≥40 kg/m^2^ were excluded because of the likelihood of extreme malnutrition and high risk of death^[26–28]^ or very severe obesity,^[29]^ respectively. All recruited subjects answered a questionnaire about their lifestyle characteristics. After age restriction and exclusion of subjects with incomplete data, we enrolled 39,524 apparently healthy subjects (26,975 men and 12,549 women), aged 40 to 79 years.

### 2.2 Measurement of parameters

Anthropometric and laboratory tests were conducted in the early morning after an overnight fast. The BMI was calculated from objectively measured weight and height according to the formula weight (kg)/height (m^2^). Subjects were divided into 6 categories based on BMI: ≤18.9; 19.0 to 20.9; 21.0 to 22.9; 23.0 to 24.9; 25.0 to 26.9; and ≥27.0 kg/m^2^, as described elsewhere.^[24]^ We took into consideration that the proposed World Health Organization (WHO) BMI cutoff points for overweight and obesity for Asian populations should be lower (≥23.0 and ≥27.5 kg/m^2^, respectively) than Western populations.^[30]^ Since the proportion of subjects classified as underweight (<18.5 kg/m^2^) or obese (≥30.0 kg/m^2^) was lower than 5% (3.6% and 4.5%, respectively), we rounded up the low and high BMI cutoffs to 19 and 27 kg/m^2^ (5.6% and 15.6%, respectively).

Routine blood parameters were measured with standard methods using Hitachi autoanalyzers (Tokyo, Japan) at the Saitama Health Promotion Corporation. Glycated hemoglobin (HbA1c) results based on the Japan Diabetes Society (JDS) were converted to the National Glycohemoglobin Standardization Program (NGSP) HbA1c units using the officially certified formula HbA1c (NGSP) (%) = 1.02 × JDS (%) + 0.25%.^[31]^ The blood tests included biochemical measurements of aspartate aminotransferase (AST) and alanine aminotransferase (ALT), γ-glutamyl transferase (GGT), white blood cell (WBC) counts, triglyceride, and high-density lipoprotein (HDL) cholesterol.

### 2.3 Questionnaire of symptoms, lifestyles, diet, and past histories

The questionnaire consisted of 32 lists of factors for evaluating health condition (Table 1). Subjects were able to select multiple symptoms without restriction. A predisposition for the common cold was expressed as self-reported susceptibility to common cold (SPCC), which corresponds to a positive response for “Predisposition to having common cold,” in the list (No. 5). To assess gastrointestinal dysfunctions, the presence or absence of gastric complaints, diarrhea, and constipation were evaluated by the results of selection No. 2, 7, and 24, respectively.

**Table 1:**
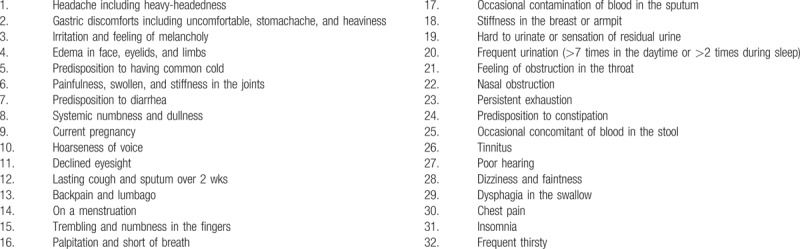
Questionnaire of self-reported symptoms (multiple choices allowed).

In addition to the symptom questionnaire (32 lists), subjects were also asked to complete a form to record the history of cardiovascular disease (including stroke), pharmacotherapies (hypertension, diabetes, or dyslipidemia), alcohol consumption (none or occasional, 1–3 times/wk, 4–6 times/wk, or daily, and the amount of ethanol (g)/1 time), smoking status (none, past, or current). Data were also collected on the Brinkman index^[32]^ of the number of cigarettes per day × years), and regular exercise (≥30 minutes per episode; none, occasional, once/wk, or at least twice/wk). Meal frequency per day, nutritional balance of diet, frequency of taking a snack, and refraining from excess salt were also assessed in a degree dependent manner. Self-reported sleep duration per night, which was obtained as a response to a simple question, was divided into 5 categories (≤5, 6, 7, 8, and ≥9 hours) according to previous studies that used a 7 hours reference duration of sleep.^[33,34]^

## 3 Statistical analysis

Differences in continuous and categorical variables between groups with or without SPCC were evaluated using *t*-test and *χ*^2^-test, respectively. Differences in the proportion of SPCC between the 6 BMI categories and between subjects with or without intestinal dysfunction (gastric complaints concomitant with constipation or diarrhea) were tested by analysis of variance (ANOVA) with post hoc tests (Bonferroni test). Multivariate logistic regression models were used to evaluate associations of SPCC with clinical categories and variables, which were sequentially adjusted for relevant confounding factors. Logistic regression models yielded odds ratios (ORs) and 95% confidence intervals (95% CIs). Statistical analyses were performed using Stat-view 5.0 and SAS-EG (SAS Institute; Cary, NC). A *P*-value <.05 was considered significant.

## 4 Results

Table 2 shows the clinical characteristics of subjects according to the absence or presence of SPCC. There was no significant difference in most clinical parameters including age, BMI, HbA1c, prevalence of men, and current smokers between the 2 groups. By contrast, WBC counts and serum ALT were significantly higher in subjects with SPCC compared with those without SPCC, whereas serum HDL-C and duration of sleep were lower. Additionally, the prevalence of having headache, cough/sputum for over 2 weeks, gastric discomforts, diarrhea, and constipation (Table 1) were higher in subjects with SPCC (all, *P* < .0001). Besides the finding that consumption of alcohol per one time was significantly lower in subjects with SPCC (*P* = .006), frequent alcohol drinkers were less prevalent in subjects with SPCC (*P* < .0001). Furthermore, persons who regularly exercised were less prevalent in subjects with SPCC (*P* < .0001), whereas those with undesirable behaviors concerning meal frequency, meal balance, and taking a snack were more prevalent (all, *P* < .0001).

**Table 2:**
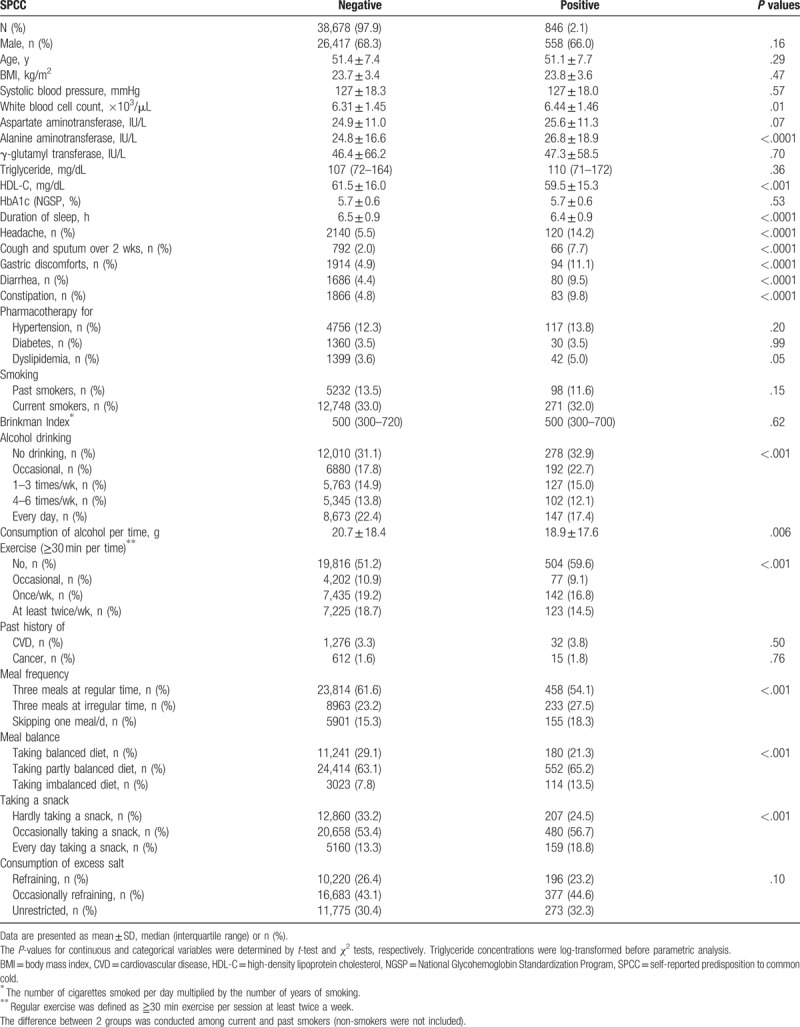
Characteristics of subjects according to the absence or presence of SPCC.

**Figure 1:**
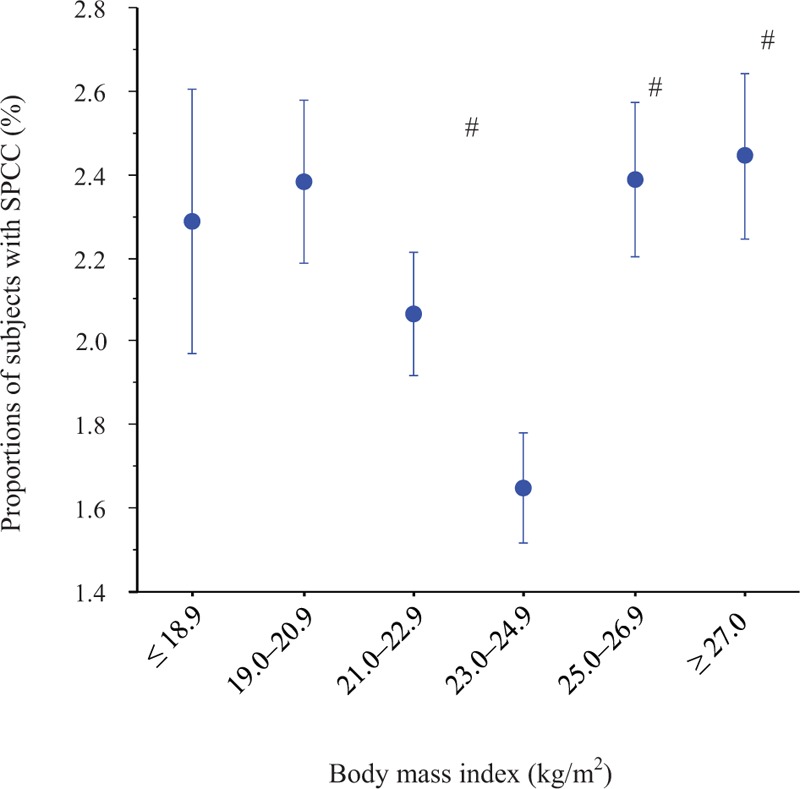
Proportions of subjects with SPCC stratified by BMI categories. The symbol in the middle of each bar is the percentage of subjects with SPCC. The vertical bar represents the s.e. when SPCC is numbered as 1 and non-SPCC as 0. ^**#**^ v.s. BMI of 23.0 to 24.9 kg/m^2^ (Bonferroni test, *P* < .0033). The number of subjects was 2230, 5961, 9098, 9355, 6702, and 6178 for the BMI categories of ≤18.9, 19.0 to 20.9, 21.0 to 22.9, 23.0 to 24.9, 25.0 to 26.9, and ≥27.0 kg/m^2^, respectively. BMI = body mass index, SPCC = self-reported predisposition to common cold.

**Figure 2:**
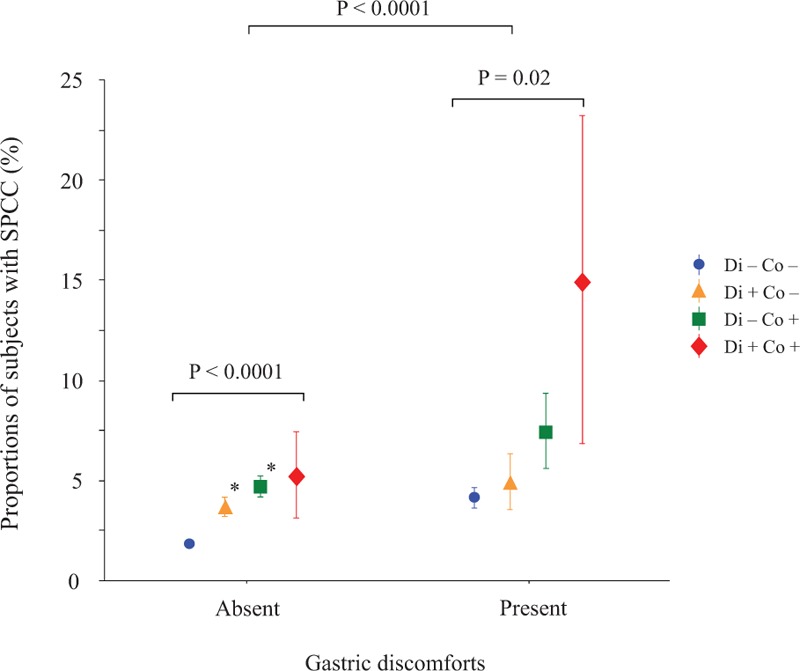
Proportions of subjects with SPCC stratified by gastric discomfort and intestinal dysfunctions. The symbol in the middle of each bar is the percentage of subjects with SPCC. The vertical bar represents the s.e. when SPCC is numbered as 1 and non-SPCC as 0. Overall, proportions of subjects with SPCC were significantly higher in subjects with gastric discomfort than those without (*P* < .0001, *t*-test). The proportions of subjects with SPCC were increased across the increasing intestinal dysfunctions (diarrhea alone, constipation alone, and both diarrhea and constipation) in those with and without gastric discomfort (*P* = .02 and *P* < .0001, ANOVA, respectively). ^**#**^ v.s. absent of intestinal dysfunctions (Bonferroni test, *P* < .0033). The number of subjects was 34,383, 1628, 1404, and 101 in the group without gastric discomforts and 1547, 200, 241, and 20 in the group with gastric discomforts for intestinal dysfunctions (none, diarrhea alone, constipation alone, and both diarrhea and constipation), respectively.Co = constipation, Di = diarrhea, SPCC = self-reported predisposition to common cold.

The relationship between the proportions of SPCC and BMI groups showed a U-shaped curve with a nadir of the BMI at 23.0 to 24.9 kg/m^2^ (Fig. 1). Compared with the BMI of 23.0 to 24.9 kg/m^2^, the BMI groups of 19.0 to 20.9, 25.0 to 26.9, and ≥27.0 kg/m^2^ had significantly higher proportions of SPCC (Bonferroni test). Figure 2 shows the proportions of SPCC according to gastric discomforts and concomitant intestinal dysfunctions (diarrhea and constipation). The SPCC proportions were significantly higher in subjects with gastric discomforts (*P* < .0001). When subjects were further divided into those with and without gastric discomforts, we observed a significant increase in SPCC across the concomitant of intestinal dysfunctions (intact, diarrhea, constipation, and both diarrhea/constipation) in both those without gastric discomforts (*P* < .0001, ANOVA) and those with (*P* = .02, ANOVA).

Table 3 shows the results of logistic regression analysis. Headache and cough/sputum lasting over 2 weeks were significantly associated with SPCC even after adjustment for all relevant confounders (Model 2). Compared with the BMI of 23.0 to 24.9 kg/m^2^, the other BMI categories were significantly associated with SPCC. However, after full adjustment for relevant confounders, the BMI of ≥27.0 kg/m^2^ was not significantly associated with SPCC. Simultaneously, compared with 7 hours duration of sleep, short duration of sleep (≤5 hours) was significantly associated with SPCC. Gastric discomforts, diarrhea, constipation, and both diarrhea/constipation together were all independently and significantly associated with SPCC regardless of adjustment for confounders. An imbalanced diet and taking a snack were also associated with SRCC in a degree dependent manner, whereas both meal frequency and consumption of excess salt were not associated with SPCC (data not shown).

**Table 3:**
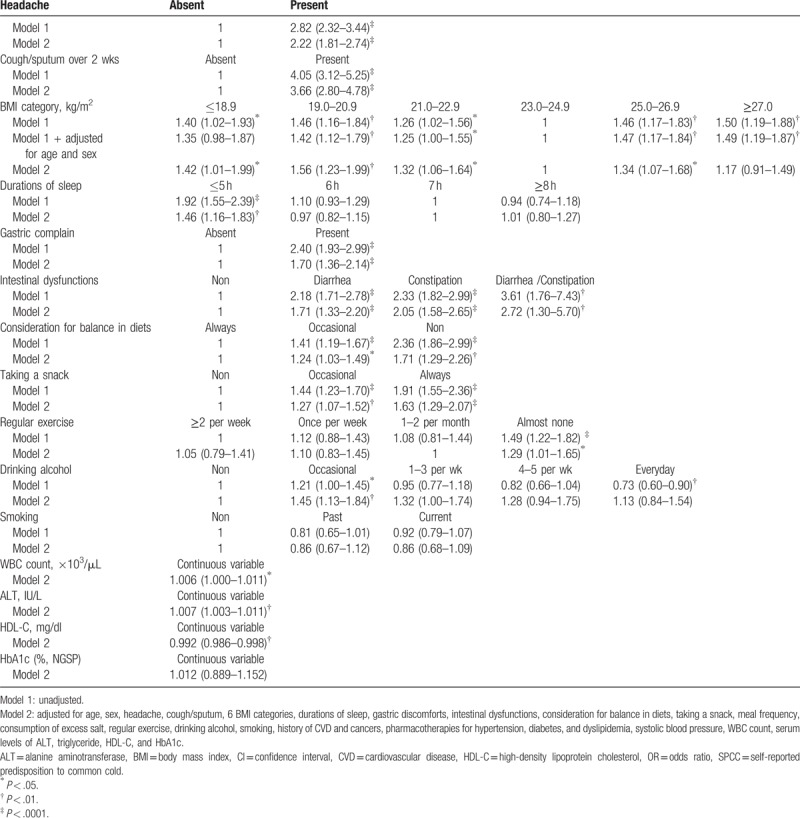
ORs (95% CIs) of potential factors for positive SPCC.

Compared with no drinking of alcohol, occasional drinking was associated with SPCC, although consumption of alcohol per se seemed to be inversely associated with SPCC albeit statistically insignificant (OR 0.89, 95% CI, 0.78–1.01, *P* = .07). Furthermore, compared with frequent exercise (≥2 times per week), no-exercise (almost none) was significantly associated with SPCC. However, after adjustment for confounders, compared with low frequent exercise (twice per month), no-exercise was marginally associated with SPCC (Model 2). After full adjustment for confounders, the associations of SPCC with WBC count, serum ALT, serum HDL-C (inversely) remained significant, whereas age, sex, systolic blood pressure, HbA1c, and pharmacotherapies for hypertension, diabetes, and dyslipidemia were consistently not associated with SPCC.

## 5 Discussion

Integrated studies investigating the associations between various ordinary factors in daily life and the common cold have not been reported to the best of our knowledge. This may be the first study that investigated the relationship between SPCC and the many factors that range from symptoms to lifestyles and cardiometabolic risks. Our study of middle-aged and elderly Japanese people demonstrated that various factors and symptoms, including BMI, duration of sleep, alcohol drinking, smoking, intestinal dysfunctions, exercise, diets, and some biochemical parameters, may be simultaneously and independently associated with SPCC. Of note, most of these factors are classified into acquired conditions that could be ameliorated by interventions or pharmacotherapies. Indeed, there may be uncertainty regarding the efficacy of interventions for preventing the common cold in the general population, probably because of its widespread morbidity and spontaneous occurrence.^[35]^ However, Ball et al^[36]^ have shown that the common cold constitution exists in children (lower interferon-gamma titers). Therefore, similar constitution might exist in adults and play key roles in the susceptibility to the common cold. This was the main purpose of our study and we discuss individually each category concerned as follows.

### 5.1 BMI

In 1992, the Japan Society for the Study of Obesity determined that the standard body weight is “a weight equivalent to BMI 22.0 kg/m^2^”^[37]^ based on the clinical evidence of the lowest morbidity for many common diseases, including lung, heart, gastrointestinal, renal, and liver disease, diabetes, and hypertension in the Japanese population.^[38,39]^ However, the BMI of 23.0 to 24.9 kg/m^2^ is slightly higher than the provisional standard BMI of 22.0 kg/m^2^ in Japan. Several studies suggest that both underweight and obesity are associated with increased risk of respiratory tract infection.^[20]^ Since the standard BMI of 22.0 kg/m^2^ has been based on the results of common diseases, which is not specific for viral infections, the optimal BMI for prevention of the common cold may be slightly higher. Intriguingly, the highest BMI category (≥27.0 kg/m^2^), obesity, was not associated with SPCC after adjustment for confounding factors, which suggests that rather than obesity itself, factors potentially related to obesity, such as shorter sleep, undesirable diets, infrequent exercise, low HDL-C, high ALT, and WBC count, and gastrointestinal dysfunctions, may have more influence on the sensitivity of the common cold.

### 5.2 Duration of sleep

Shorter sleep duration compared with the reference duration of sleep (7 hours) has been reported to be associated with increased susceptibility to the common cold,^[40,41]^ which is entirely consistent with our results. We observed that short sleep duration of ≤5 hours was persistently associated with SPCC throughout the analysis, confirming that adequate duration of sleep is a pivotal factor for the prevention of the common cold. Although some clinical studies have shown the possibility that longer sleep duration also contributes to increased risk for obesity, type 2 diabetes and high mortality,^[42–44]^ the association between SPCC and longer sleep duration over 7 hours was not observed in our study.

### 5.3 Alcohol drinking

We found that no drinking of alcohol compared with occasional (infrequent) drinking, but not frequent or daily drinking, was associated with SPCC, although the underlying mechanism is unknown. Sadakane et al^[45]^ have shown in a prospective cohort study that men who drank only on special occasions had the highest all-cause mortality, regardless of alcohol intake per drinking session. In addition, Naimi et al^[46]^ have shown that among those with low average alcohol consumption, infrequent drinkers drink more during drinking days and have unfavorable risk profiles compared with more frequent drinkers. Furthermore, Ouchi et al^[3]^ have shown that high frequency of alcohol intake (daily alcohol drinking) was significantly associated with low prevalence of common cold in Japanese men, whereas high amount of alcohol intake (>35.8 g/day) was not. Taken together, a plausible explanation for our results concerning alcohol is that frequent alcohol consumption may exert as protective action against the common cold, which might be applicable among drinkers (from occasional to daily drinkers) but not nondrinkers. Nevertheless, other overlooked confounding factors that can be detected, for instance, by a diet or behavioral survey, may interfere with the outcomes.

### 5.4 Gastrointestinal symptoms and diets

Chronic constipation and diarrhea have been associated with gut microbiota and immune function.^[47,48]^ Therefore, we may expect associations between SPCC and these gastrointestinal dysfunctions, although no clinical studies have previously investigated these associations. Our study showed that gastric discomfort was associated with SPCC regardless of intestinal dysfunctions (constipation and diarrhea), which suggests that both colon and gastric function probably interfere with common cold infections. Balanced diets that include adequate amounts of vitamins and minerals, such as vitamin C and zinc, have been shown to reduce the incidence of common cold symptoms concomitant with the common cold.^[4,49–51]^ In children, taking a snack has been reported as a link to recurrent respiratory infection.^[52,53]^ Likewise, taking a snack may be associated with higher incidental risk for the common cold in adults, albeit the underlying mechanism is unknown. In our study, we found that meal frequency was not associated with SPCC, which indicates that total energy or the nutrients taken per day may be important for SPCC or immune function.

### 5.5 Habitual exercise

We found that no-exercise was significantly associated with SPCC compared with infrequent exercise (1–2 per month), whereas mild to moderately frequent exercise was not, suggesting that high frequency exercise (≥2 times per week) might not have a protective effect for the common cold in the general population. Nevertheless, the type (aerobic or anaerobic), amount and the duration of exercise were unknown in our study. Although some studies have shown a potential protective effect of exercise on the common cold,^[54,55]^ the evidence is limited. Instead, regular or rigorous exercise increases both the incidence and severity of upper respiratory illness.^[56]^ Other concomitant factors such as nutritional status and other lifestyle factors, may synergically contribute to the incidence of the common cold.

### 5.6 Smoking

As reported previously,^[57–60]^ we expected a significant association between SPCC and smoking. In our study, we divided non-current smokers into no-smokers and past smokers. In addition, we also considered the number of cigarettes smoked per day and the period smoked (Brinkman index). However, neither of current and past smokers and Brinkman index were associated with SPCC, although current smoking was persistently associated with lasting cough and sputum. In previous studies,^[57,58]^ subjects were intentionally exposed to common respiratory viruses and smokers developed infections at higher rates, which suggest that smokers may be more susceptible to the common cold once they are exposed to a pathogen. Nevertheless, in actual daily life, it is possible that smokers pay attention in avoiding exposure to a pathogen and reducing the incident risk of the common cold.

### 5.7 Biochemical parameters and pharmacotherapies related to cardiometabolic diseases

In this study, WBC and ALT were positively associated and HDL-C was inversely associated with SPCC. This we expected because these variables are likely to relate to the immune function and the condition of the infection.^[61–64]^ However, it is unknown why HbA1c and pharmacotherapy for diabetes were not associated with SPCC. Several studies have revealed that patients with diabetes are at increased risk of severe influenza disease,^[17,19]^ whereas it is ambiguous whether these patients are at increased risk of the common cold. Similar to the null association between obesity (≥27.0 kg/m^2^) and SPCC mentioned earlier, diabetes related conditions including an imbalanced diet, taking a snack, short duration of sleep, infrequent exercise, and gastrointestinal dysfunctions, might have a greater influence than the diabetes per se on the susceptibility to the common cold.

### 5.8 Limitations

First, our study was cross-sectional in nature and did not allow us to conclude a cause–effect relationship between SPCC and the conditions and parameters investigated, which could lead to inverse causality without careful interpretations. Second, the assessments of predisposition to the common cold relied solely on the one subjective outcome measurement. Nevertheless, it was not feasible to evaluate precisely the predisposition to the common cold in a large epidemiological study, because virological testing is time- and cost-consuming and a validated questionnaire for the common cold has not been established. However, we showed that SPCC was significantly associated with concomitant symptoms of the common cold (headache and lasting cough/sputum),^[1,4]^ suggesting that SPCC may roughly reflect the predisposition to the common cold. Third, the diagnostic technique in the clinic or hospital could not confirm that SPCC did not include influenza infections.

## 6 Conclusion

Our results demonstrated that multifactorial conditions and parameters might be simultaneously associated with the predisposition to the common cold, suggesting that the suppression of causative factors and the promotion of protective factors could prevent the common cold, which could improve quality of life, work productivity, and economic loss. Prospective studies including detailed common cold questionnaire and measurements, and randomized control trials may be required to confirm our observations.

## Acknowledgments

The authors thank Kenneth Tieszen, PhD, from Edanz Group (www.edanzediting.com/ac) for editing a draft of this manuscript.

## Author contributions

MS and KN designed the overall study analyzed data, and wrote the manuscript draft. KS identified eligible subjects from the database at Saitama Health Promotion Corporation. MS, TI, RH, and KN discussed the results and literature. All authors read and approved the final manuscript.

**Conceptualization:** Kaname Suwa, Kei Nakajima.

**Data curation:** Michi Shibata, Taizo Iwane, Ryoko Higuchi, Kaname Suwa, Kei Nakajima.

**Formal analysis:** Michi Shibata, Taizo Iwane, Ryoko Higuchi, Kei Nakajima.

**Investigation:** Michi Shibata, Ryoko Higuchi, Kei Nakajima.

**Methodology:** Kaname Suwa.

**Project administration:** Kei Nakajima.

**Resources:** Ryoko Higuchi, Kei Nakajima.

**Software:** Taizo Iwane.

**Supervision:** Kei Nakajima.

**Validation:** Taizo Iwane, Kei Nakajima.

**Writing – original draft:** Kei Nakajima.
